# The Campaign against Cancer

**Published:** 1915-04

**Authors:** 


**A Monthly Review of Medicine and Surgery**

EDITOR AND PUBLISHER, DR. A. L. BENEDICT, 228 Summer St., corner of Elmwood Ave., Buffalo, (Address for all communications. Please make personal and telephone calls before 1 P. M.)

Third, in age, on the western continent. Independent, but supports professional organization. News limited mainly to Western N. Y. and Penn.

Subscription $2.00 a year; $3.00 for two years in advance. Changes and errors in address or failure or duplication of delivery, should be reported immediately.

Yearly Volume 70. APRIL, 1915 No. 9

### The Campaign Against Cancer.

The Commission on Cancer of the Medical Society of the State of Pennsylvania has asked us to co-operate, along with other medical journals, in this work, by devoting space to the subject in the July issue. We, therefore, urge our readers to submit articles on the subject and, in accordance with our general policy, it is scarcely necessary to state that such articles should present the matter in a scientific and practical way and should avoid, so far as possible, matters of common knowledge or ignorance.

This may be an opportune time to reiterate certain opinions that may be considered heterodox, even pessimistic. No one, either from the journalistic or the practitioners' point of view can consider the cancer problem as more deserving of investigation than the editor. Cancer accounts for the great majority of those most unfortunate cases in his practice, in which the physician fights a losing battle. He has pointed out in the Medical Record of April 18, 1914, that cancer kills more than 18 per cent. of all women who die between the ages of 50 and 54; more than 9 per cent. of all men who die ten years later; that cancer ranks as almost the greatest if not the greatest cause of death between the ages of 45 and 75, when human life ought to have its highest social value.

In spite of this appreciation of the magnitude of the problem, there are certain aspects in which we do not quite agree with expressions of opinion at present popular. First of all, we think that the medical profession, private philanthropists and official dispensers of public philanthropy, should look the difficulties of the problem squarely in the face. Cancer has been definitely recognized by the medical profession, even by the laity, for several thousand years. So far as empiric and "practical" experience goes, nothing has been accomplished in all this time, in the way of radical prophylaxis or cure, except a very limited success from destructive methods. Strictly scientific bases for attacking the problem date back not much more than a quarter of a century and it must be acknowledged that, thus far, we have not even come to a substantial agreement as to either the essential nature, cause, or method of cure of cancer although it would be unjust not to assert that great progress has been made, even with regard to technical improvements in the attempts at radical cure. On the one hand, it must be admitted that there is not much reason for hoping that the cancer problem will be solved, even to a partial but significant degree in the immediate future, on the other hand, the magnitude of the problem justifies the expenditure of the greatest amount of energy and money and any such expenditure will be fully justified, both on an economic and a humanitarian basis, if the problem is even partially solved in a century. There should be no remission of professional interest or of private or public support, merely because immediate practical results are not forthcoming. Indeed, the time comparison just made, makes a century, a very small estimate on which practical results from an investment should be judged.

Secondly, it should be just as definitely recognized that, to date, cancer has developed no generally accepted analogy to certain diseases for which the prophylactic and therapeutic measures can be practically separated. Various other diseases have remained even to a greater degree incurable and of unknown nature and scientific etiology, after empiric methods of prophylaxis have become pretty well established. So far as cancer is concerned, prophylaxis is limited either to the avoidance of general predisposing causes not very specifically connected with the disease or to the eradication of certain lesions —for example gastric ulcers and cicatrized cervixes—in which cancer shows a moderate tendency to develop.

Thirdly, it may be pretty definitely stated as practically true, that the treatment of cancer is at present limited to the destruction of a local lesion, not extending to any general biologic reaction, and this fact renders the therapeutics, in a sense prophylactic. In other words, while we cannot apply any hygienic regimen or biologic method to ward off the disease, we must apply our diagnostic and therapeutic methods of radical relief at an early stage of the disease.

Under these circumstances, we question whether mere agitation of the subject can accomplish much. It is necessary to publish articles on cancer often enough to prevent neglect but we can see no benefit to be derived from the accumulation of a mass of literature which does not further professional knowledge.

Neither do we feel that the time is ripe for a systematic enlistment of popular interest, until the profession can do something more than admit its ignorance and its dissentions. It is true that prompt diagnosis and even more prompt treatment—for, barring external and some peculiarly significant internal cancers, treatment must precede diagnosis to have any radical value—will increase the percentage of cures by destruction of foci. But a popular campaign along these lines should, we think, not be limited to cancer; partly because of the harm of arousing a general state of fear, partly because of that of making too prominent a state of professional ignorance for which only our general stage of progress can be blamed. We would urge, therefore, that any campaign to urge the laity to pay prompt attention to incipient disease, should be along broader lines. Cancer is one of the largest factors which makes such prompt recourse to diagnossi and treatment necessary but, on the other hand, it is almost if not quite the most important factor in which even such promptness will prove unavailing. Taking into consideration all ages, and all diseases, cancer mortality is merely a small part of the direful results of neglect of premonitory and admonitory symptoms.

Moreover, we think the time is ripe to protest against the reiterated charge that the medical profession itself is responsible for delays preventing radical cure of cancer, and especially against the tendency to pass this charge to the laity. That these delays occur in individual cases cannot be denied but the matter is so constantly brought before the profession that we can not understand how any physician who reads or hears discussions of the subject can be further influenced by eloquence. The man who neglects prompt attention to incipient cancer cannot, in our opinion, be reached by any means except the gradual weeding out process applied to medical education, ante and post graduate, and involving all branches of practice. In a considerable experience as consultant, we have been impressed with the rarity of neglect of attention to cases suspected of being cancerous and, that this statement may not be construed as a gallery play for popularity, it may be qualified by the criticism that, if anything, there is a professional tendency to feel tumors where none are present, to confuse with cancer, almost any other serious local involvement of certain organs or manifestations of other general processes, such as tuberculosis, and to explore, promptly from the standpoint of individual attendance, cases whose general history and physical signs pointed clearly to inoperable conditions including those in which there might be some doubt as to the existence of cancer but in which the course of the disease showed plainly that, if cancerous, it must be far beyond the stage of radical relief and in that condition in which any major operation would almost certainly result in speedy death.

There is a further reason why we think lay propaganda against cancer are untimely. Fifteen years ago, we could have gone to the laity with an insistance on prompt opportunities for diagnosis and prompt operation, even in advance of diagnosis. We should have had to admit no alternative treatment, except caustics in certain superficial growths and for these, no practical advantage but rather greater pain and greater uncertainty. At present, various emanations and modifications of cataphoretic methods deserve serious consideration, especially in inoperable cases, yet they involve dangers of omission and commission. Speaking judicially, and for the profession generally, we can neither accept nor condemn these methods nor lay down rules as to choice as against operation or as to choice among what may generally be considered alternative methods. When we consider that a comparatively few years will probably enable pretty definite statements to be made, as regards choice of destructive methods, and that there is even some ground to expect a more general specific biologic method of treatment, it seems unwise to the highest degree to subject the laity to a conflict of enthusiasts and conservatives, to pave the way for an attack of quacks equipped with more or less genuine emanation apparatus and the like or, at best, to make a public confession of therapeutic vacillation on top of a frank avowal of general ignorance of scientific nature and cause of the disease.

